# Microsaccades reflect the dynamics of misdirected attention in magic

**DOI:** 10.16910/jemr.12.6.7

**Published:** 2019-06-28

**Authors:** Anthony S. Barnhart, Francisco M. Costela, Susana Martinez-Conde, Stephen L. Macknik, Stephen D. Goldinger

**Affiliations:** Carthage College, Kenosha, Wisconsin, USA; Schepens Eye Research Institute, Harvard Medical School, Boston, Massachusetts, USA; SUNY Downstate Medical Center, Brooklyn, New York, USA; Arizona State University, Tempe, Arizona, USA

**Keywords:** Eye movements, eye tracking, microsaccades, attention, divided attention, magic

## Abstract

The methods of magicians provide powerful tools for enhancing the ecological validity of laboratory studies of attention. The current research borrows a technique from magic to explore the relationship between microsaccades and covert attention under near-natural viewing conditions. We monitored participants’ eye movements as they viewed a magic trick where a coin placed beneath a napkin vanishes and reappears beneath another napkin. Many participants fail to see the coin move from one location to the other the first time around, thanks to the magician’s misdirection. However, previous research was unable to distinguish whether or not participants were fooled based on their eye movements. Here, we set out to determine if microsaccades may provide a window into the efficacy of the magician’s misdirection. In a multi-trial setting, participants monitored the location of the coin (which changed positions in half of the trials), while engaging in a delayed match-to-sample task at a different spatial location. Microsaccades onset times varied with task difficulty, and microsaccade directions indexed the locus of covert attention. Our combined results indicate that microsaccades may be a useful metric of covert attentional processes in applied and ecologically valid settings.

## Introduction

In the last decade, exploration of the methods and intuitions of magicians has gained traction as a route to understanding the mind [1, 2, 3], with some researchers going so far as to speak of *neuromagic* as a new field of scientific enquiry [4, 5], or call for the development of a formal *science of magic* [6, 7, 8]. While this movement has its detractors [9, 10], the scientific exploration of magic has benefited multiple research areas, including the study of motion perception [11, 12], change blindness [13, 14], problem solving [15, 16], decision making [17, 18, 19], attitude change [20, 21], motor control [22, 23], temporal attention [24, 25], and eyewitness memory [26].

Arguably, magic’s greatest influence on cognitive neuroscience has been seen in the study of eye movements and attention [25, 27, 28], where magicians’ methods can be implemented to enhance the ecological validity of experimental paradigms. Kuhn and Tatler [29] were among the first to use magic in laboratory studies of inattentional blindness, the tendency for people to miss salient events when engaged in an attentionally-demanding task. Participants watched a magician (Kuhn) vanish a cigarette and a cigarette lighter while their eye motions were tracked. Because the cigarette was visibly dropped in the magician’s lap, participants should have detected the method for the vanish, had they deployed their attention appropriately. However, the magician dropped the cigarette at the same moment as he revealed that the *cigarette lighter* had also vanished, thereby producing a high rate of inattentional blindness for the falling cigarette. The vast majority of participants (90%) failed to detect this highly salient event, even though it took place right in front of them. Interestingly, participants’ fixation positions at the start of the cigarette’s fall did not differ as a function of whether they did or did not detect the drop, indicating that it was not overt, but covert attentional deployment, that differed between the two groups. Kuhn, Tatler, Findlay, and Cole [30] replicated this outcome with the magic trick shown on video rather than live, though rates of inattentional blindness dropped to 43%. Even so, the participants’ susceptibility to inattentional blindness remained unrelated to their fixation positions at the start of the drop. 

More recently, Barnhart and Goldinger [31] studied inattentional blindness with a different magic trick. Participants viewed a video of a magician (Barnhart) while their eye movements were tracked (see video at https://youtu.be/wkTsl0qZp7g). The magician placed a silver coin on one side of a placemat, and then covered the coin with a napkin. Next, he placed an identical napkin on the opposite side of the placemat. The magician then positioned inverted cups on top of each napkin, after showing the inside of each empty cup to the camera. At this point in time, participants were queried on the location of the coin. Appropriately deployed attention would have allowed participants to detect that, while the magician showed the inside of the first cup to the camera, the coin visibly slid from its initial position under one of the napkins to a different location, beneath the other napkin. Yet, 55% of participants failed to detect the sliding coin. In agreement with the previous reports by Kuhn and colleagues [29, 30], fixation positions at the midpoint of the coin’s movement were unrelated to detection of the moving coin (although participants who detected the coin were more likely to fixate the space through which the coin moved during the greater critical period when the coin was visibly moving). Again, these findings suggested that covert attentional mechanisms are critical to inattentional blindness. This conclusion is consistent with traditional inattentional blindness research, which has likewise failed to find significant differences in overt attentional deployment between participants who experience detection failures and those who do not [32, 33].

### Microsaccades as an index of covert attention

By definition, *covert* attentional mechanisms are not accompanied by externally noticeable signals, seemingly making it impossible to generate online predictions about where a person’s attention is placed. Instead, one must make such inferences based on subsequent behavior. Despite this, studies conducted over the past several years have provided substantial evidence that *microsaccades,* a class of fixational eye movements, may reliably point to the location of covert attention, making the covert a bit more overt [34, 35, 36, 37] (see Martinez-Conde, Otero-Millan, & Macknik [38] for a review). 

Microsaccades are operationalized as small-amplitude (<1-2 deg) binocular eye movements occurring 1-2 times per second during attempted fixation [38, 39]. Early researchers proposed that microsaccades primarily served to correct fixation errors [40] and counteract adaptation [41]. While there is empirical support for these assertions, recent research suggests myriad roles for microsaccades in perception and attention (see Martinez-Conde et al. [38] for a review), including sampling information from information-rich regions in a visual scene [42], preventing and counteracting perceptual fading during fixation [43, 44], correcting gaze-position errors [45], facilitating extraction of fine details from a small region of space [46] and aiding resolution of perceptual ambiguity [47].

Hafed and Clark [35] were the first to find that microsaccades were biased in the direction of covert attention, in an exogenous orienting task. A similar outcome was observed shortly thereafter by Engbert and Kliegl [34]. However, the findings were initially met with criticism – Horowitz, Fine, Fencsik, Yurgenson, and Wolfe [48] used a cueing task similar to that of Engbert and Kliegl, but their analyses focused primarily on instances where microsaccade directions *deviated* from the cued location. If microsaccades serve as an index of covert attention, they argued, then “erroneous” microsaccades away from the cue on *invalid* trials should lead to faster response times (RTs) than erroneous microsaccades on valid trials. In essence, participants would accidentally attend the location where the target would subsequently appear, thus facilitating its detection. Horowitz et al. found no such speeding of RTs on such trials, leading them to conclude “no systematic relation between microsaccade direction (…) and attention” (p. 362). A rebuttal to Horowitz et al. showed that the mapping between microsaccade direction and target location did account for significant variance in RTs [49], and a later experiment from the same researchers, with a more refined design, showed a substantially stronger correlation between microsaccade direction and RTs [50].

While the existence of *some* relationship between attention and microsaccades is now generally well accepted, much of the work done to assess this relationship has focused on exogenous attentional capture, rather than endogenous attentional control. In one exception, a study monitored the perception of stimuli appearing in locations that were congruent or incongruent with spontaneously-generated microsaccades, and found enhanced perceptual accuracy in congruent locations [37]. The extent to which this research elicited endogenous variations in attention is unknown, as participants were not actively attending to any stimuli in particular when microsaccades occurred. 

The effects of task difficulty on microsaccade dynamics have also been reported. Pastukhov and Braun [51] found an inverse relationship between microsaccade rates and task difficulty in a visual attention task. Siegenthaler et al. [52] similarly found that microsaccade rates decreased, and microsaccade magnitudes increased, with task difficulty during mental arithmetic.

Here we set out to explore the relationship between microsaccades and attention within an endogenous attention task with varied levels of difficulty, and to do so in a real-world scenario, under near-natural viewing conditions. We adapted the inattentional blindness design from Barnhart and Goldinger [31] for this purpose, so that participants were aware that the coin might move from its initial position. In the original experiment from Barnhart and Goldinger, participants were not aware that the coin could move, or that they were about to witness a magic trick, leading to high rates of inattentional blindness. In the current experiment, participants were made aware that the coin could move from its original location. Thus, the coin was no longer an inattentional blindness stimulus. The video was not presented as a magic trick. Participants were asked to engage in dual tasks in every trial: a) to monitor the coin location, and b) to engage in a delayed match-to-sample (MTS) task with stimuli presented within the cups. Performing optimally on both tasks required participants to divide their attention between two vertically-aligned locations: the first cup, shown near the top of the screen, and the coin, which slid horizontally along the bottom of the screen in half the trials. Half of participants were allowed to freely move their gaze for the duration of each video, and half were required to maintain fixation on a spatial position that coincided with the location of the MTS stimuli. 

Laubrock et al. [50] noted that most microsaccades are oriented horizontally, and therefore may not be ideal to detect vertically-divided attention. However, few experiments have made vertically-divided attention a requirement, and so this potential limitation in microsaccadic dynamics has not been systematically tested. Our task demanded the vertical division of attention during a circumscribed time window. If microsaccades index covert attention along the vertical axis, then there should be a clear increase in vertical microsaccades during this critical period. In order to have adequate sensitivity to detect microsaccade directions with dynamic stimuli, we instantiated a between-subjects manipulation, wherein half of participants had to maintain fixation on the location of the MTS stimuli throughout each trial (constrained viewing condition), and the other half were allowed to view the stimuli freely (free-viewing condition). We anticipated that microsaccade dynamics related to features of the task would be most apparent during constrained viewing, but we also expected many of the same effects to appear during free viewing, as task demands would necessitate that participants fixate the MTS stimuli during the critical period when the MTS sample is presented and the coin may be moving.

Further, because our design required effective perception only (i.e. without planning for a button-press), it also allowed us to address the possibility that the microsaccade biases observed in prior research during attentional cuing tasks reflected mere motor planning [48], rather than covert attentional deployment. If this motor planning hypothesis is correct, then microsaccades in the present experiment should have no relationship (either directional or temporal) with the task at hand.

## Method

### Participants

A total of 61 Arizona State University undergraduates (23 female) participated for course credit (28 in the free-viewing condition; 33 in the constrained viewing condition). Sample size was dictated by the number of participants who could be recruited during a single ASU semester, and was consistent with sample sizes from previous research in this area [34]. All participants had normal or contact lens-corrected vision. Protocols were approved by the Arizona State University Institutional Review Board and were in compliance with the Declaration of Helsinki.

### Stimuli

Stimuli consisted of four videos previously used by Barnhart and Goldinger [31] (see supplementary materials). In each video, a magician (Barnhart) places a coin (an American 50 cent piece) at one of two positions on a dark placemat. The coin is then covered with a napkin, and a second napkin is placed on the opposite side of the mat. Then, the magician shows the inside of a paper cup to the camera before placing it over the first napkin. Next, he repeats this same action for a second cup, which he subsequently places over the second napkin. In two of the videos, the coin visibly moves from under the first napkin to under the second napkin (either from left to right, or from right to left), at the same time as the magician shows the inside of the first cup to the camera. The coin remains visible, during its horizontal displacement, for an average of 550ms (or 16.5 frames at 30fps). In the other two videos, the coin stays in its original location beneath the first napkin (on either the left side or the right side of the placemat) and does not move across the mat. All videos had a duration of 22 seconds, except for the no-movement video with the coin starting under the right napkin, which had a duration of 21 seconds.

**Figure 1.**
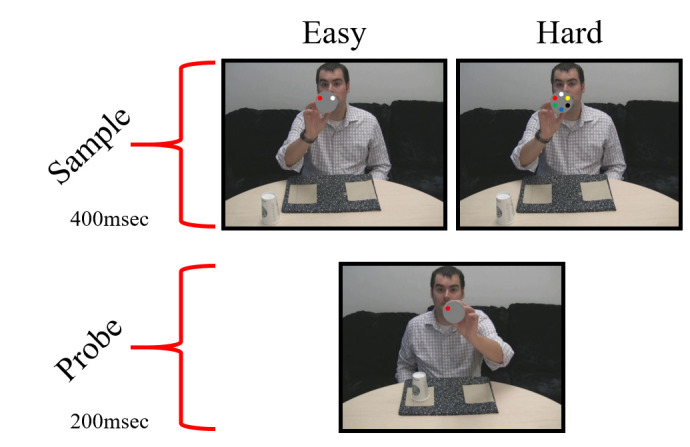
Examples of MTS stimuli in the Easy and Hard conditions. The probe should elicit a “match” response.

Adobe^®^ Photoshop^®^ software was used to superimpose stimuli for the delayed match-to-sample (MTS) task over relevant frames of each video, which were then compiled back into video files using Adobe^®^ Premiere Pro^®^. The stimuli were embedded within the video files to ensure the timing of stimuli relative to events in the video. The *sample* stimuli consisted of a circular array of colored dots: either six dots in the hard MTS condition or 2 dots in the easy MTS condition (Figure 1). The sample onset was concurrent with the point in each video where the inside of the first cup is shown to the camera. The sample was visible for 400ms (or 10 video frames at 30fps). The *probe* stimulus consisted of a similar circular display with a single colored dot placed at one of the six dot positions. The probe stimulus was overlaid upon the video at the point when the inside of the second cup is displayed to the camera, and was visible for 200ms (or 5 frames at 30fps). The MTS stimuli were positioned at a stationary location over the centroid of the cup’s trajectory through the relevant frames in each video. On average, the delay between sample offset and probe onset was 3625ms (σ = 271ms).

For each of the four video conditions, 144 MTS versions were created crossing probe color (6 levels; white, yellow, black, blue, green, or red) by probe position (6 levels; 2, 4, 6, 8, 10, and 12 o’clock) by MTS ease (2 dots vs. 6 dots) by MTS condition (match vs. no-match). The colors and positions of distractor dots were selected randomly for each video during stimulus creation, as were the colors of individual probe dots on the no-match MTS trials. In total, a pool of 576 video files was created from which trial stimuli were sampled.

### Apparatus

Participants’ heads were stabilized with a chin rest while their binocular eye movements were monitored at 500Hz via an SR Research Eye-Link 1000 tracker with a spatial resolution of 0.01°. Stimuli were presented and responses were collected using SR Research Experiment Builder software running on a Dell Optiplex 755 PC (2.66 GHz, 3.25 GB RAM) with a 20-inch NEC FE21111 CRT display (60Hz refresh; 1024x768 resolution). 

### Procedure

After obtaining informed consent, participants’ gaze was calibrated on the eye-tracker, via a nine-point calibration procedure. Participants repeated the calibration procedure until their average error fell below 0.5° of visual angle and no errors exceeded 1° visual angle. This calibration procedure was repeated after every twelve trials in the experiment, and every trial started with a drift correction screen that required participants to press the spacebar while fixating a central dot.

Participants were informed that, on each trial, they would see the insides of two cups shown to the camera: 

*Inside the first cup, you’ll see a collection of colored dots. Your job is to remember the arrangement of the dots. Specifically, you need to try to remember which color dot is in each position. In the second cup, you’ll see only one colored dot. Your job is to decide whether this colored dot appeared in this position within the first cup.*

Participants were also directed to monitor the ending location of the coin on each trial. The experiment began with five practice trials followed by 48 trials counterbalancing Coin Starting Position (left, right), Coin Movement Condition (move, no move), MTS Ease (easy, hard), and MTS Response (match, no match). After each video, participants both reported whether the probe matched the sample and where the coin was at the end of the trial, by pressing the “z” or “m” keyboard keys. No other variables were manipulated, and no other responses were collected. Participants received immediate feedback on their accuracy for each judgment.

**Figure 2.**
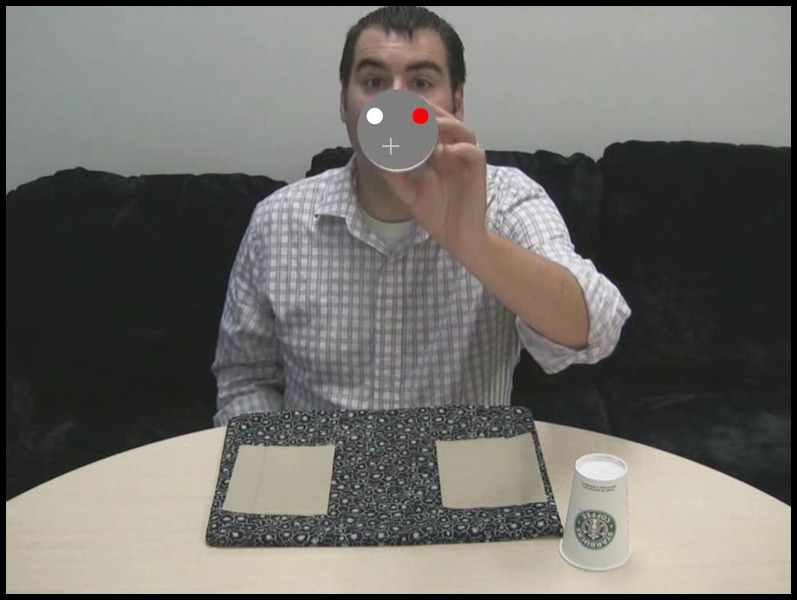
Screen shot depicting the fixation target present for participants in the constrained viewing condition.

Throughout the experiment, half the participants were allowed to move their eyes freely (free-viewing condition). The other half of participants were required to maintain fixation within an invisible 130 x 130 pixel box placed over the centroid of the MTS stimuli across videos (constrained viewing condition). If a participant’s eyes left the box for more than 600ms, a tone sounded until they resumed fixation. A grey crossbar (i.e. a fixation target) was overlaid on the video to facilitate fixation maintenance (see Figure 2 and the constrained viewing video in Supplemental Materials). No other variables were manipulated or measured.

### Eye Movement Analyses

One participant was excluded from the constrained viewing condition due to a tracking failure. Eye movement analyses were limited to participants who erred on more than five MTS trials. These exclusions left 26 participants in the constrained viewing condition and 24 in the free-viewing condition. Constrained viewing trials where participants failed to maintain fixation were dropped from analyses.

Saccades were identified with a modified version of the algorithm developed by Engbert and Kliegl [34, 53, 54, 55, 56], with λ = 5 (used to obtain the velocity threshold) and a minimum saccadic duration of 6 ms. Microsaccades were defined as saccades with magnitude < 1.5 deg in both eyes [39, 43, 57, 58, 59, 60]. To calculate microsaccade properties such as magnitude and peak velocity we averaged the values for the right and left eyes. 

For each subject, correlations between microsaccade onsets and sample onsets were smoothed using a Savitzky-Golay filter of order 1 and a window size of 151 ms [43]. Average correlations are the average of the smoothed correlations. 

## Results

### Task Accuracy

Participants’ MTS performance was less accurate on the hard trials (69%) than on the easy trials (93%), thus validating our task difficulty manipulation. A mixed model ANOVA on MTS accuracy rates with within-subject factors Ease (easy, hard) and Coin Movement (move, no move) and between-subjects factor Viewing Condition (free, constrained) revealed no significant effects other than Ease (*F*(1, 58) = 220.60, *p* < .001, η^2^_p_ = .79). The same analysis produced no reliable effects on coin detection accuracy, which was at ceiling (M=94%).

### Microsaccades during constrained viewing

**Figure 3.**
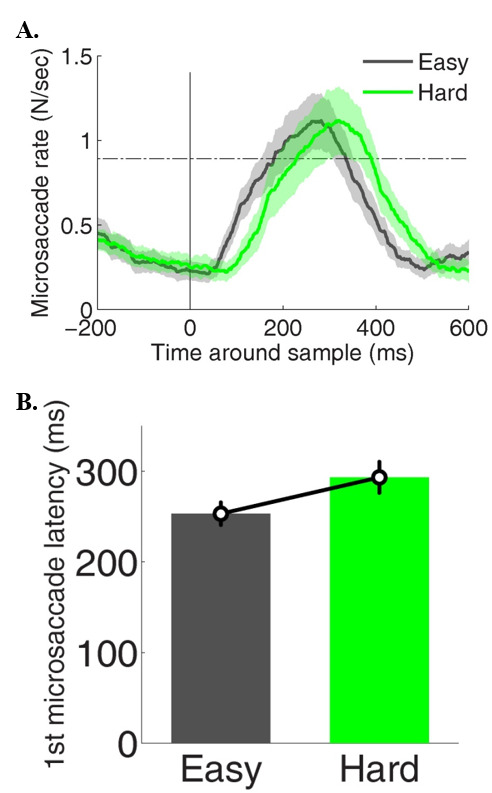
Timecourse of microsaccades during the easy and hard MTS tasks. (A) Microsaccade rates around MTS stimuli onset (gray vertical line). Horizontal line corresponds to the baseline. Shaded areas indicate S.E.M across subjects. (B) Average latency of the first microsaccade after each MTS stimuli onset. Error bars indicate S.E.M across subjects.

*Time-course Analyses.* Although overall microsaccade rates did not differ significantly across hard and easy MTS trials, the time-course of microsaccade onsets revealed considerable discrepancies as a function of MTS task difficulty (Figure 3A). First, microsaccade latencies after the onset of the MTS stimuli were different in easy vs. hard task conditions (p < 0.01), being significantly lower during the easy task (253 ms +/-12) than during the hard task (293 ms +/- 17) (Figure 3B).

**Figure 4.**
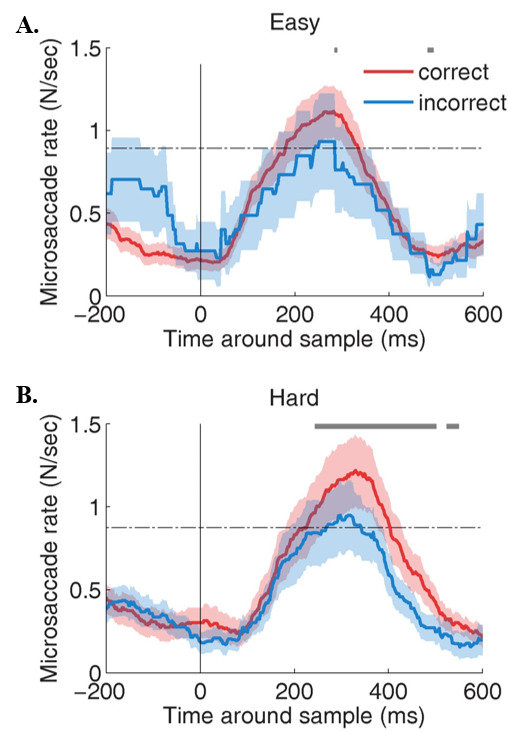
Timecourse of microsaccades in the easy (A) and hard (B) MTS task conditions. Gray line indicates significance for one-tailed paired t-tests using Bonferroni Correction. α= 0.01. Shaded areas indicate S.E.M across subjects.

Microsaccade rates differed significantly for correct vs. incorrect trials in the hard MTS task, but not in the easy task (Figure 4A). Specifically, microsaccade rates during the 600 ms after sample onset were significantly higher for correct (*p*= 0.02; average rate = 0.61 +/- 0.06) than for incorrect trials (average rate = 0.54 +/- 0.05) in the hard MTS task condition (Figure 4B).

We examined the time-course of microsaccade dynamics around the critical viewing period (i.e. starting with the onset of the MTS stimuli), when participants had to divide their attention between the MTS stimuli and the coin.Microsaccade rates were higher in the 2s-interval before the onset of the critical period (0.71 Hz) than afterwards (0.38 Hz), and gradually returned to baseline after ~2 s (not shown). We also examined microsaccadic dynamics as a function of coin movement. Microsaccade rates were significantly higher during the 2 seconds before the coin motion onset (1.01 ± 0.06 Hz) than during the 2 seconds immediately afterwards (0.59 ± 0.05 Hz; two tailed paired t-test: *p* < 10^-8^). In trials where the coin remained still, microsaccade rates during the equivalent periods followed a similar pattern, being significantly higher before the point in time when coin motion onset would normally occur (0.99 ± 0.06 Hz) than afterwards (0.63 ± 0.06 Hz); two tailed paired t-test: *p* < 10^-6^. One possible explanation for such dynamic difference in microsaccade rates in both types of trials could be that participants were aware that the coin might move at a specific point in time (i.e. the coin motion onset), and were accordingly modulating their fixation behavior around this time window.

**Figure 5.**
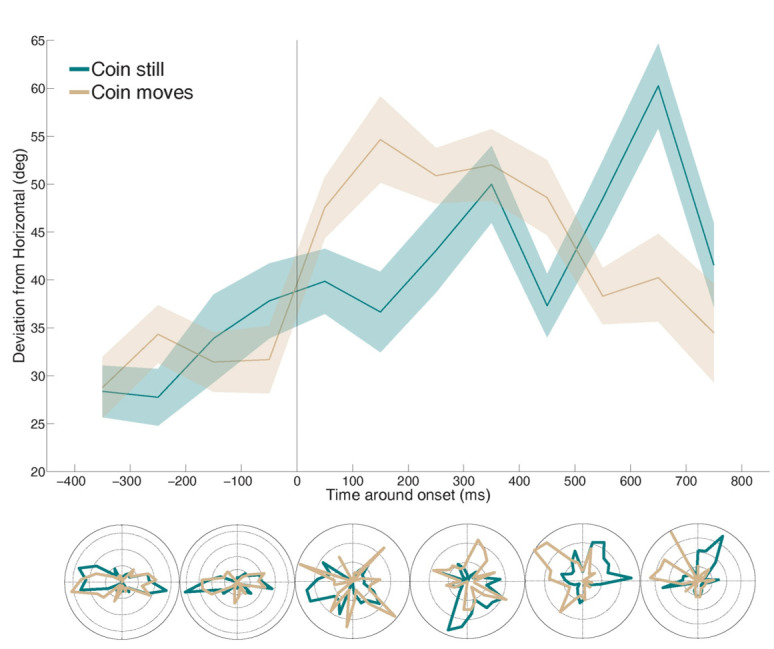
Average deviation from horizontal direction for microsaccades produced around the presentation of MTS stimuli. Polar histograms are shown in 200 ms intervals.

*Direction Analyses.*Next, we examined the microsaccadic deviation from horizontal (dfh) direction around the presentation of the MTS stimuli, analyzing microsaccades in 200-ms bins. To create polar histograms, we transformed microsaccade directions (in degrees) to radians and calculated a histogram with 50 bins. The results of these histograms were then normalized and a Cartesian plot was created given the bins (theta) in radians and the radius (rho) from the value of the histogram for each bin. Microsaccade dfh did not differ significantly across hard and easy MTS trials. However, microsaccade dfh did vary about the critical period when participants needed to divide their attention between two vertically-oriented points. Microsaccades were mainly horizontal before the critical period onset, but then shifted to vertical within the critical viewing period (Figure 5).

In the moving coin condition, microsaccade direction became significantly more vertical after the coin started moving (47.97° ± 2° dfh) than before it did (30.68° ± 2° dfh; two-tailed paired *t*-test: *p* < 10^-5^). There was a specific time period where microsaccades were mostly vertical, between 0 and 600 ms. We found a significant effect in the deviation from horizontal between bins (ANOVA repeated-measures analysis *F*(5,60)=4.09; *p*<.01). 

Similarly, in the still coin condition, microsaccade direction became significantly more vertical after the start of the critical period (41.42° ± 2° dfh) than before it (31.34° ± 1° dfh; two-tailed paired *t*-test: *p* = 0.05). The deviation from horizontal was also significantly different between bins (ANOVA repeated-measures analysis, *F*(5,55)=5.24; *p*<.001). Vertical microsaccades were most prominent in this condition during the last bin examined (600-800), presumably corresponding with an expectation that the coin might reappear.

**Figure 6.**
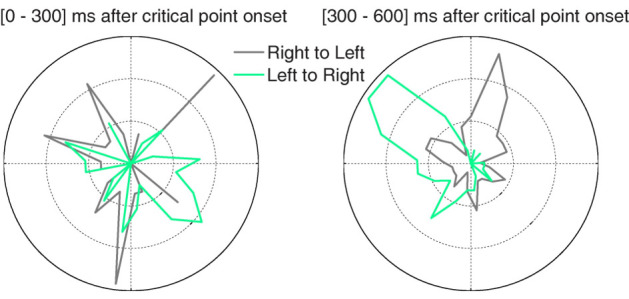
Polar histograms for microsaccades during the critical period, as the coin was moving. Green: trials where the coin moved from left to right. Grey: trials where the coin moved from right to left

Finally, we asked whether microsaccade directions might be biased towards the coin’s movement direction (i.e. either from left to right or from right to left). We calculated polar histograms for the moving coin condition, [0-300] ms and [300-600] ms after critical point onset (Figure 6). There were significant differences in the deviation from vertical (*F*(1, 15)=8.01, *p*=.01). In both time bins, microsaccades showed a strong directional bias towards the downward direction, presumably anticipating the final position of the coin movement (rightward in the ‘left to right’ condition and leftward in the ‘right to left’ condition). This is consistent with the participants’ instructions, which directed them to monitor the ending location of the coin on each trial. In contrast, microsaccades had a strong upward component in the second interval, likely indicating that participants returned their gaze to the MTS stimuli location (rightward in the ‘right to left’ condition and leftward in the ‘left to right’ condition).

### Microsaccades during free-viewing

**Figure 7.**
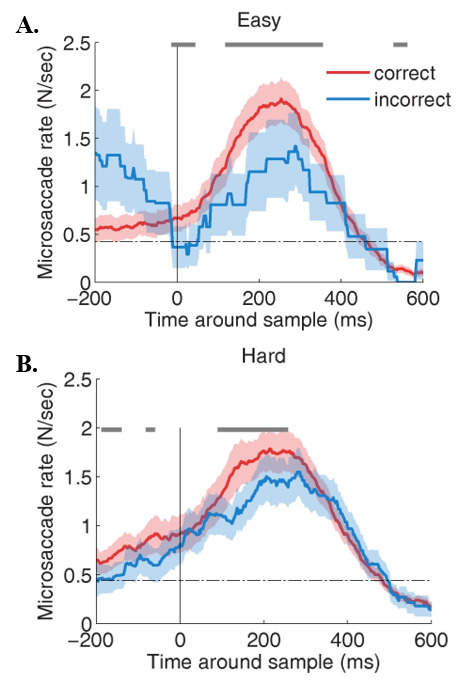
Analysis of the time course of the microsaccade between MTS response conditions for (A) easy MTS task and (B) hard MTS task. Gray line indicates significance for one-tailed paired t-tests using Bonferroni Correction. α= 0.01. Shaded areas indicate S.E.M. across subjects.

*Time-course Analyses.* As with the constrained viewing condition, the overall microsaccade rates did not differ between the easy and hard conditions. Unlike the constrained viewing condition, however, the time-course of microsaccade onsets was unaffected by MTS task difficulty. In this viewing condition, microsaccade rate predicted accuracy for both easy (Figure 7A) and hard (Figure 7B) MTS tasks.

We also examined the time-course of microsaccade dynamics around the critical viewing period, when participants had to divide their attention between the MTS stimuli and the coin. Microsaccade rates during the 2 seconds before the critical point (when the coin was still) were significantly smaller than during the 2 seconds after the critical point (0.54 ± 0.04 microsaccades/s; two tailed paired t-test: p < 10^-6^). No significant change was found in microsaccade rate with coin movement onset.

**Figure 8.**
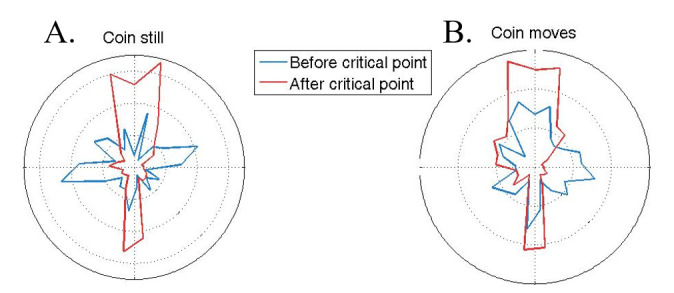
Polar histograms of microsaccades 700 ms before and 700 after the critical point onset. (A) Coin still condition. (B) Coin motion condition.

**Figure 9.**
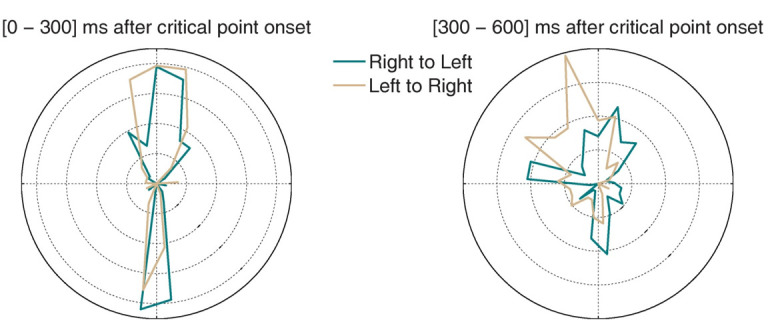
Polar histograms for microsaccades after the critical point onset. Green indicates trials where the coin moved from right to left, and yellow those where the coin moved from left to right.

*Direction Analyses*. Microsaccade dfh did not significantly differ across the easy and hard conditions. However, dfh did differ before and during the critical period. Microsaccades after the critical point were more vertical than before it. Specifically, microsaccades became significantly more vertical during the 700 ms after the critical point in trials where the coin was still (55.2° ± 1.8° deviation from horizontal), compared to the 700 ms before the critical point (36.8° ± 1.6° deviation from horizontal; two-tailed paired t-test: p < 10^-5^; Figure 8A), as well as in trials where the coin moved (53.9° ± 2° deviation from horizontal), compared to the 2 seconds before the coin movement onset (41.8° ± 2° deviation from horizontal; two-tailed paired t-test: p < 10^-4^; Figure 8B). The microsaccade direction polar histograms showed a strong vertical bias: both upward and downward in the [0-300] ms interval, and upward in the [300-600] ms interval after the critical point onset (Figure 9).

## Discussion

We found that both the time-course and direction of microsaccades varied with the spatial demands of our task, suggesting that microsaccades can serve as an index of covert attention in natural viewing conditions. When participants had to divide their attention between two vertical points, microsaccade directions shifted in both constrained- and free-viewing conditions. Prior to this critical viewing period, most microsaccades were horizontal, but when attention needed to be vertically divided to successfully accomplish the dual task, the vertical component of microsaccades increased substantially. Furthermore, in the constrained viewing condition (where participants’ gaze was spatially limited to a small visual area, making their microsaccade directions easier to discriminate), microsaccades were biased in directions that aided participants’ performance. Specifically, microsaccades directions were biased toward the endpoint of the coin movement at the beginning of the critical viewing period, and redirected back to the MTS stimulus at the end of the critical period. 

Microsaccade rates and onsets also varied as a function of viewing condition and task properties. In the free-viewing condition (and in the hard trials of the constrained viewing condition), microsaccade rates predicted accuracy in the MTS task. That is, participants who generated higher microsaccade rates while encoding the sample stimulus were more likely to respond accurately to the probe later on in the trial. This pattern supports the notion that microsaccades serve to acquire visual information from informative scene regions [44], and that they facilitate the scanning (and subsequent encoding) of small regions of space [46, 61]. We also observed clear evidence of microsaccadic inhibition that varied with task complexity. In the constrained viewing condition, the hard MTS trials resulted in longer microsaccadic latencies (for the first microsaccade produced during the critical period) than the easy MTS trials did. This finding is consistent with reports by Pasthukov and Braun [51 and Siegenthaler et al. [52] that microsaccade rates fall with task difficulty in visual [51] and non-visual [52] tasks. It is also in line with research by Valsecchi, Betta, and Turatto [62] showing prolonged microsaccadic inhibition when participants were required to encode details from an unexpected stimulus, and suggesting that microsaccadic inhibition might help reduce information loss from saccadic suppression [63].

Although the participant experiences across the free- and constrained viewing conditions were likely subjectively different, the outcomes of both conditions were remarkably similar. The consistency in the effects observed across the two conditions indicates that constraining the participants’ view (even if it resulted in a more artificial setup) had no effect on fixational eye movement quality. The salient differences between viewing conditions appeared in the timecourse analyses. Microsaccadic inhibition (as measured by microsaccadic latencies) was increased for hard MTS trials in the constrained viewing condition, but not in the free viewing condition. Although fixation patterns during the critical period were quite similar across viewing conditions, free viewing may have allowed participants to establish more optimal fixation locations, facilitating quick information extraction relative to the constrained viewing condition, thus reducing microsaccadic inhibition. The second notable difference between viewing conditions was in microsaccade rates before and during coin movement. In the constrained viewing condition, microsaccade rates were reduced at the time point when the coin *could *have been moving, regardless of whether it did move. In the free viewing condition, the opposite occurred. Microsaccade rates increased during this time period, but only during trials where the coin was still. This inconsistency may have been due to differences in motion capture of covert attention during constrained and free viewing. Boyer and Wang [64] presented evidence that motion cues capture attention to a greater extent during constrained than free viewing. The increased microsaccade rate during free viewing may have been an attempt to compensate for reduced motion capture.

One limitation of the present study is that performance on the coin detection task was at ceiling, thereby disallowing the exploration of whether microsaccades might serve as an online predictor of susceptibility to inattentional blindness. Future research should reduce the salience of the peripheral stimulus to induce greater error rates. This could be accomplished by using a smaller or less shiny coin, for example. Given that the present microsaccadic dynamics did successfully index both task difficulty and the spatial allocation of the subjects’ attention, it seems reasonable to expect that microsaccades may be a fruitful predictive metric of inattentional blindness in forthcoming studies [65].

Despite this limitation, the current work represents the first study to examine how microsaccades correlate with the perception of dynamic, real-world stimuli during the endogenous control of attention. The time-course and direction of microsaccades had clear relationships to the processing demands inherent to the task, and could not be explained as an artefact of motor planning (cf. Horowitz et al. [48]). Our combined results indicate that microsaccades are an important tool for extracting detail from a complex visual array where attention needs to pool in disparate locations. 

Finally, the present research helps to solidify the value that the art of magic can offer to the science of the mind. While the current experiment did not explicitly use magic tricks to probe awareness, as others have, it borrowed techniques from the magician’s toolbox to produce a more naturalistic experimental context, with applicability to everyday experiences. Magicians are master choreographers of attention, and their insights and techniques supply ample fodder for experimentation and theory development. We hope that future research will continue to mine the methods and principles of magicians for fruitful techniques and hypotheses that are worthy of testing. The intersection of magic and cognitive science has already proven valuable in the study of inattentional blindness and attentional capture, and it likely contains many more unexplored notions that may help accelerate the rate of cognitive and neuroscientific discovery [4, 8].

### Ethics and Conflict of Interest

The author(s) declare(s) that the contents of the article are in agreement with the ethics described in http://biblio.unibe.ch/portale/elibrary/BOP/jemr/ethics.html and that there is no conflict of interest regarding the publication of this paper. 

### Acknowledgements

This research was supported in part by NICHD Grant R01 HD075800-01 to Stephen D. Goldinger, National Science Foundation Award 1523614 to Stephen L. Macknik, and National Science Foundation Award 1734887 to Stephen L. Macknik and Susana Martinez-Conde. We thank Dr. Gustav Kuhn and an anonymous reviewer for their thoughtful comments on earlier drafts and Dr. Michael B. McCamy for valuable discussions and help with data analyses.
